# Latent profiles of sport participation motivation and their association with campus sports culture among higher vocational college students

**DOI:** 10.3389/fpsyg.2026.1950464

**Published:** 2026-10-02

**Authors:** Yiyuan Zhang, Ding Wang, Wenting Yan

**Affiliations:** Suzhou Vocational Health College, Suzhou, Jiangsu Province, China

**Keywords:** campus sports culture, higher vocational college students, latent profile analysis, motivational climate, sport participation motivation

## Abstract

**Objective:**

This study identified latent profiles of sport participation motivation among higher vocational college students and examined their associations with campus sports culture.

**Methods:**

A cross-sectional online survey was conducted in 2026 among students from three higher vocational colleges in Jiangsu and Zhejiang Provinces using convenience sampling. Campus sports culture and sport participation motivation were assessed using the Campus Sports Culture Scale and the Chinese version of the Behavioral Regulation in Exercise Questionnaire-3. Latent profile analysis was performed in Mplus 8.3, followed by chi-square tests and the R3STEP three-step procedure to examine demographic differences and associations between campus sports culture and profile membership while accounting for classification uncertainty.

**Results:**

Four motivational profiles were identified: the Low-to-Moderate Flat Profile (5.8%), Autonomous Orientation Profile (33.1%), High Controlled–Amotivation Profile (8.4%), and Moderate Mixed-Motivation Profile (52.6%). The Moderate Mixed-Motivation Profile was the largest subgroup. With the Low-to-Moderate Flat Profile as the reference category, higher overall campus sports culture scores were associated with greater odds of membership in the Autonomous Orientation, High Controlled–Amotivation, and Moderate Mixed-Motivation Profiles, with the strongest association observed for the Autonomous Orientation Profile. At the dimensional level, material culture was associated with all three profiles, whereas spiritual and behavioral culture were associated with the Autonomous Orientation and Moderate Mixed-Motivation Profiles. Institutional culture was not significantly associated with profile membership.

**Conclusion:**

Sport participation motivation among higher vocational college students showed substantial heterogeneity, with mixed motivational regulation being the most common pattern. Campus sports culture was differentially associated with motivational profile membership, particularly with profiles containing stronger autonomous regulation. These findings suggest that accessible sport resources, an active participation climate, and recognition of sport value may be relevant considerations when developing profile-sensitive physical activity programs in higher vocational colleges.

## 1 Background

Sport participation motivation (SPM) refers to the psychological drive that initiates, sustains, and regulates engagement in sport and physical activity. It reflects why students participate, how they value sport, and whether they are willing to maintain regular participation (Deng et al., 2023). SPM is closely associated with physical activity frequency, participation quality, and patterns of engagement, making it an important psychological correlate of sport behavior. Previous studies have linked SPM to health-related goals, intrinsic interest, enjoyment, perceived competence, peer interaction, achievement striving, and external evaluation, underscoring its multidimensional and context-sensitive nature (Abas et al., 2023; Yang et al., 2024). Among higher vocational college students, SPM may show distinctive characteristics because of their learning modes, career-oriented educational goals, and campus life structures. Rather than a single continuous variable, motivation in this group may appear as different configurations or latent subtypes (Gao and Mohamad, 2025). Some students may be driven mainly by health promotion and enjoyment, whereas others may participate because of course requirements, peer influence, institutional expectations, or external evaluation. These patterns may correspond to differences in sport engagement, participation quality, and behavioral persistence (Gao and Mohamad, 2025). Therefore, SPM should be examined not only by overall mean scores but also by its internal heterogeneity. Latent profile analysis (LPA) offers a person-centered approach to identifying homogeneous subgroups based on responses across motivational dimensions, thereby revealing motivational patterns that may be obscured by traditional variable-centered analyses. Self-Determination Theory (SDT) provides the theoretical basis for understanding these motivational configurations. Within this framework, behavioral regulation ranges from amotivation and more controlled forms of regulation to increasingly self-determined forms, including identified regulation, integrated regulation, and intrinsic motivation (Teixeira et al., 2012). These forms of regulation are not necessarily mutually exclusive and may coexist within the same individual. This makes a person-centered approach particularly useful for examining how different forms of sport participation motivation are combined among higher vocational college students. Higher vocational college students (HVCS) are an important subgroup in China’s higher education system, and their sport participation has both public health and vocational education significance. In 2024, enrollment in regular undergraduate, vocational undergraduate, and higher vocational college programs reached 38.9126 million in China; vocational undergraduate and higher vocational college students accounted for 18.0544 million, or approximately 46.4% of the total higher education population (Ministry of Education of the People’s Republic of China, 2025). Compared with regular undergraduates, HVCS are more strongly oriented toward career training, practical skill development, and employment preparation. Their academic schedules, internship arrangements, and campus experiences may therefore be particularly relevant to the way sport participation motivation is organized in this population (Li, M., et al., 2024; Gao and Mohamad, 2025). Insufficient physical activity among college students has become a growing concern. The 2023 Chinese College Student Health Tracking Survey showed that only 30.2% of students had engaged in moderate-to-vigorous physical activity in the previous month (Li et al., 2024b), while another survey across 31 provinces found that 77.6% mainly participated in low-intensity physical activity (Li et al., 2024a). International evidence is similar, with 65.0% of Brazilian undergraduates reported as physically inactive (de Santana et al., 2023). These findings suggest that inadequate sport and physical activity participation is a broader issue, but the low level of moderate-to-vigorous activity among Chinese college students indicates particular concern. For HVCS, professional learning, skill-based training, internships, and employment pressure may further limit the time and energy available for sport participation. Their engagement may also be especially sensitive to institutional arrangements, campus resources, peer support, and perceived physical demands of future occupations (Gao et al., 2025). Regular sport participation can support physical fitness, stress regulation, and occupational readiness, whereas intensive academic and employment demands may reduce sustained participation (Teuber et al., 2024). Therefore, identifying latent profiles of sport participation motivation among HVCS is important for clarifying the psychological basis of insufficient sport participation and for developing stratified, targeted physical activity promotion strategies in higher vocational colleges.

Campus sports culture (CSC) refers to the material environment, institutional arrangements, behavioral practices, and value orientations that develop around school-based sport and physical education. It encompasses facilities and activity spaces, curriculum and management systems, patterns of participation, and the value attached to sport by teachers and students (Gu et al., 2010). Rather than representing a single environmental feature, CSC captures several aspects of the campus context that may be differently related to students’ motivation for sport. Previous research has linked physical activity and exercise motivation to environmental resources, peer and social support, organized opportunities for participation, perceived competence, autonomy support, and the broader campus sport climate (Abula et al., 2020; Brown et al., 2024; Li et al., 2025; Murphy et al., 2024; Scroggs et al., 2025; Wang et al., 2024). A two-wave study of Chinese college students, for example, found that perceptions of the campus sport environment were associated with physical fitness through peer support and perceived physical literacy (Song and Yang, 2026). Person-centered evidence also indicates that exercise motivation cannot be adequately represented by a single average score. Gao et al. (2025) identified five motivational profiles among college students and found differences in sport companionship and physical education participation across profiles. Related studies have linked sport preference, exercise self-efficacy, supportive social contexts, and motivational processes to exercise motivation and physical activity (Hu and Liu, 2025; Liang et al., 2025; Sheng et al., 2025; Sun et al., 2025). However, relatively little is known about how different forms of sport participation motivation coexist among higher vocational college students or whether specific dimensions of CSC are associated with these motivational configurations. Most previous studies have focused on general university samples or examined motivation using variable-centered approaches. To address this gap, the present study used LPA to identify naturally occurring profiles across the six BREQ-3 regulations and subsequently examined demographic differences and the associations of overall and dimension-specific CSC with profile membership. Given the cross-sectional design, these analyses were intended to characterize patterns of association rather than establish temporal or causal relationships.

## 2 Participants and methods

### 2.1 Participants

A convenience sampling approach was used to recruit students from three higher vocational colleges located in Nanjing and Suzhou, Jiangsu Province, and Hangzhou, Zhejiang Province, between February and April 2026. A total of 2,166 students completed an online questionnaire. Ethical and consent procedures were adapted to participants’ age. Participants aged 18 years or older provided informed consent before beginning the questionnaire. For participants younger than 18 years, informed consent was obtained from a parent or legal guardian, together with assent from the participant, before participation. Only participants for whom the appropriate consent procedures had been completed were permitted to proceed to the questionnaire. Participation was entirely voluntary, with no academic credit or financial compensation provided, and participants could withdraw at any time without penalty. After excluding 141 questionnaires with evident patterned responding or missing data, 2,025 valid questionnaires were retained, yielding an effective response rate of 93.5%. The final sample comprised 905 male and 1,120 female students, including 839 first-year, 672 second-year, and 514 third-year students. The mean age was 18.94 ± 1.66 years. The study protocol was approved by the Ethics Committee of Suzhou Vocational Health College (Approval No. SWYXLL202601).

### 2.2 Measures

#### 2.2.1 Campus sports culture scale

Campus sports culture (CSC) was assessed using the Campus Sports Culture Scale originally developed by Gu et al. (2010) and subsequently revised by Zhang (2022). The scale consists of 16 items across four dimensions: material culture, institutional culture, spiritual culture, and behavioral culture, with four items in each dimension. All items are rated on a 5-point Likert scale. In the present sample, Cronbach’s α coefficients for material, institutional, spiritual, and behavioral culture were 0.842, 0.840, 0.846, and 0.838, respectively. A four-factor confirmatory factor analysis (CFA) using the weighted least squares mean- and variance-adjusted estimator (WLSMV) showed a good fit to the data: *χ*^2^ (98) = 233.825, CFI = 0.995, TLI = 0.994, RMSEA = 0.026 (95% CI: 0.022–0.030), and SRMR = 0.019, supporting the proposed four-dimensional structure.

#### 2.2.2 Behavioral regulation in exercise questionnaire-3 (BREQ-3)

Sport participation motivation was assessed using the Chinese college student version of the Behavioral Regulation in Exercise Questionnaire-3 (BREQ-3). The BREQ-3 builds on the BREQ-2 developed by Markland and Tobin (2004), which incorporated amotivation, and further includes integrated regulation items developed by Wilson et al. (2006). The version used in the present study was adapted and examined among Chinese college students by Xu and Fan (2018). The full BREQ-3 contains 24 items representing six forms of behavioral regulation along the self-determination continuum: amotivation, external regulation, introjected regulation, identified regulation, integrated regulation, and intrinsic motivation. All items are rated on a 5-point Likert scale. Based on the item analysis and measurement recommendations reported for the Chinese college student version, Item 19 of the identified regulation subscale was excluded from scoring in the present study. Accordingly, identified regulation was calculated as the mean of the remaining three items, whereas each of the other five dimensions was calculated as the mean of four items. Raw subscale totals were not used. Instead, item means were calculated for all six dimensions, placing each motivational indicator on the same 1–5 metric despite the difference in the number of scored items. The indicators were not standardized before analysis. These six unstandardized subscale means were used as the manifest indicators in the latent profile analysis, and no overall motivation score was calculated. Cronbach’s α coefficients for amotivation, external regulation, introjected regulation, identified regulation, integrated regulation, and intrinsic motivation were 0.841, 0.806, 0.788, 0.779, 0.826, and 0.847, respectively. A six-factor CFA based on this scoring structure showed a good fit to the present data: *χ*^2^ (215) = 475.098, CFI = 0.991, TLI = 0.990, RMSEA = 0.024 (95% CI: 0.021–0.027), and SRMR = 0.022. By contrast, retaining Item 19 resulted in substantially poorer fit (CFI = 0.800, TLI = 0.767, RMSEA = 0.113, SRMR = 0.078), providing additional support for the scoring approach used in the present study.

### 2.3 Statistical analysis

Latent profile analysis (LPA) was conducted in Mplus using the six BREQ-3 dimension scores—amotivation, external regulation, introjected regulation, identified regulation, integrated regulation, and intrinsic motivation—as manifest indicators. Models with one to five profiles were estimated sequentially. The optimal number of profiles was determined by jointly considering the Akaike information criterion (AIC), Bayesian information criterion (BIC), sample-size-adjusted BIC (aBIC), Lo–Mendell–Rubin likelihood ratio test (LMR), entropy, average posterior probabilities, profile size, and theoretical interpretability. After the final profile solution had been established, the R3STEP three-step procedure was used to examine associations of sex, grade, and campus sports culture with latent profile membership. This procedure estimates multinomial logistic regression coefficients while accounting for classification uncertainty, thereby avoiding the assumption that most-likely profile membership is an error-free observed outcome. Accordingly, all multinomial logistic regression estimates reported in this study were obtained using R3STEP rather than by treating most-likely profile assignment as an error-free observed outcome. The Low-to-Moderate Flat Profile was used as the reference category. Sex was coded with males as the reference group, and grade was dummy-coded with first-year students as the reference group. Most-likely profile assignment was used only for descriptive cross-tabulations and *χ*^2^ comparisons of demographic distributions. Categorical variables were compared using *χ*^2^ tests, with Cramér’s V reported to quantify the strength of association. Adjusted standardized residuals were additionally examined to identify the cells contributing to significant overall associations; absolute residuals greater than 1.96 were interpreted as indicating observed frequencies that differed from expected frequencies at the two-sided 0.05 level, with positive and negative values indicating overrepresentation and underrepresentation, respectively. Before the four dimensions of campus sports culture were entered simultaneously into the R3STEP model, their intercorrelations and variance inflation factors (VIFs) were examined to assess potential multicollinearity. All four dimensions were subsequently entered together so that their associations with profile membership could be estimated while adjusting for the other CSC dimensions and demographic covariates. All continuous campus sports culture predictors were entered using their original, unstandardized scores; accordingly, the reported odds ratios represent the change in the odds of profile membership associated with a one-point increase in the corresponding predictor. All statistical tests were two-sided, with *p* < 0.05 considered statistically significant.

## 3 Results

### 3.1 Latent profile analysis and profile naming of sport participation motivation among higher vocational college students

Models with one to five profiles were estimated using the six BREQ-3 dimension scores, each ranging from 1 to 5, as manifest indicators (Table 1). AIC, BIC, and aBIC decreased progressively as the number of profiles increased. The four-profile model yielded an entropy of 0.832, which was higher than that of both the three-profile model (0.767) and the five-profile model (0.775). The LMR test further indicated that the four-profile solution provided a significantly better fit than the three-profile solution (*p* < 0.001). Although the information criteria continued to decrease and the LMR test remained significant for the five-profile solution, entropy declined, and the additional profile largely overlapped with existing motivational patterns without providing a clearly distinct theoretical interpretation. Considering classification accuracy, profile size, model parsimony, and interpretability within the self-determination theory continuum, the four-profile solution was therefore retained. The model-estimated proportions of C1–C4 were 5.8, 33.1, 8.4, and 52.6%, respectively. Based on most-likely posterior probability assignment, 107 students (5.3%) were classified into C1, 663 (32.7%) into C2, 170 (8.4%) into C3, and 1,085 (53.6%) into C4. The average posterior probabilities of correct classification ranged from 0.851 to 0.924 (Table 2). The model-estimated means and standard errors for all six BREQ-3 dimensions across the four profiles are reported numerically in Table 3 and displayed graphically in Figure 1. Based on these profile-specific patterns, C1 was labeled the Low-to-Moderate Flat Profile, C2 the Autonomous Orientation Profile, C3 the High Controlled–Amotivation Profile, and C4 the Moderate Mixed-Motivation Profile. These profiles represent relative motivational patterns within the present sample and should not be interpreted as clinical or diagnostic categories.

**Table 1:** Fit indices for latent profile models of sport participation motivation among higher vocational college students.

Number of profiles	Number of parameters	AIC	BIC	aBIC	Entropy	LMR (P)	BLRT (P)	Profile proportions
1	12	31,552.891	31,620.251	31,582.126	–			1.000
2	19	29,852.762	29,959.415	29,899.051	0.715	<0.001	<0.001	0.369/0.631
3	26	29,080.716	29,226.663	29,144.059	0.767	0.003	<0.001	0.310/0.076/0.614
4	33	28,596.459	28,781.698	28,676.855	0.832	<0.001	<0.001	0.058/0.331/0.084/0.526
5	40	28,323.566	28,548.099	28,421.017	0.775	<0.001	<0.001	0.054/0.102/0.314/0.072/0.458

**Table 2:** Average posterior probabilities of membership across the four latent profiles.

Assigned profile	Membership probability			
	C1	C2	C3	C4
C1	0.924	0.000	0.024	0.051
C2	0.001	0.920	0.000	0.079
C3	0.022	0.000	0.851	0.127
C4	0.014	0.056	0.022	0.909

**Table 3:** Model-estimated means and standard errors of the BREQ-3 dimensions across the four latent profiles.

Dimension	Low-to-Moderate Flat Profile	Autonomous Orientation Profile	High Controlled–Amotivation Profile	Moderate Mixed-Motivation Profile
Amotivation	2.323 (0.110)	2.067 (0.040)	4.062 (0.080)	3.244 (0.030)
External regulation	2.251 (0.096)	2.294 (0.040)	4.006 (0.086)	3.297 (0.029)
Introjected regulation	2.340 (0.100)	2.806 (0.038)	3.570 (0.092)	3.316 (0.029)
Identified regulation	2.209 (0.133)	4.205 (0.029)	2.644 (0.097)	3.478 (0.030)
Integrated regulation	2.227 (0.095)	4.196 (0.029)	2.624 (0.076)	3.509 (0.032)
Intrinsic motivation	2.348 (0.108)	4.345 (0.026)	2.358 (0.067)	3.580 (0.033)

Values are model-estimated means, with standard errors in parentheses. The six BREQ-3 indicators were calculated as unstandardized subscale item means and therefore remained on the common original 1–5 response metric.

**Figure 1:**
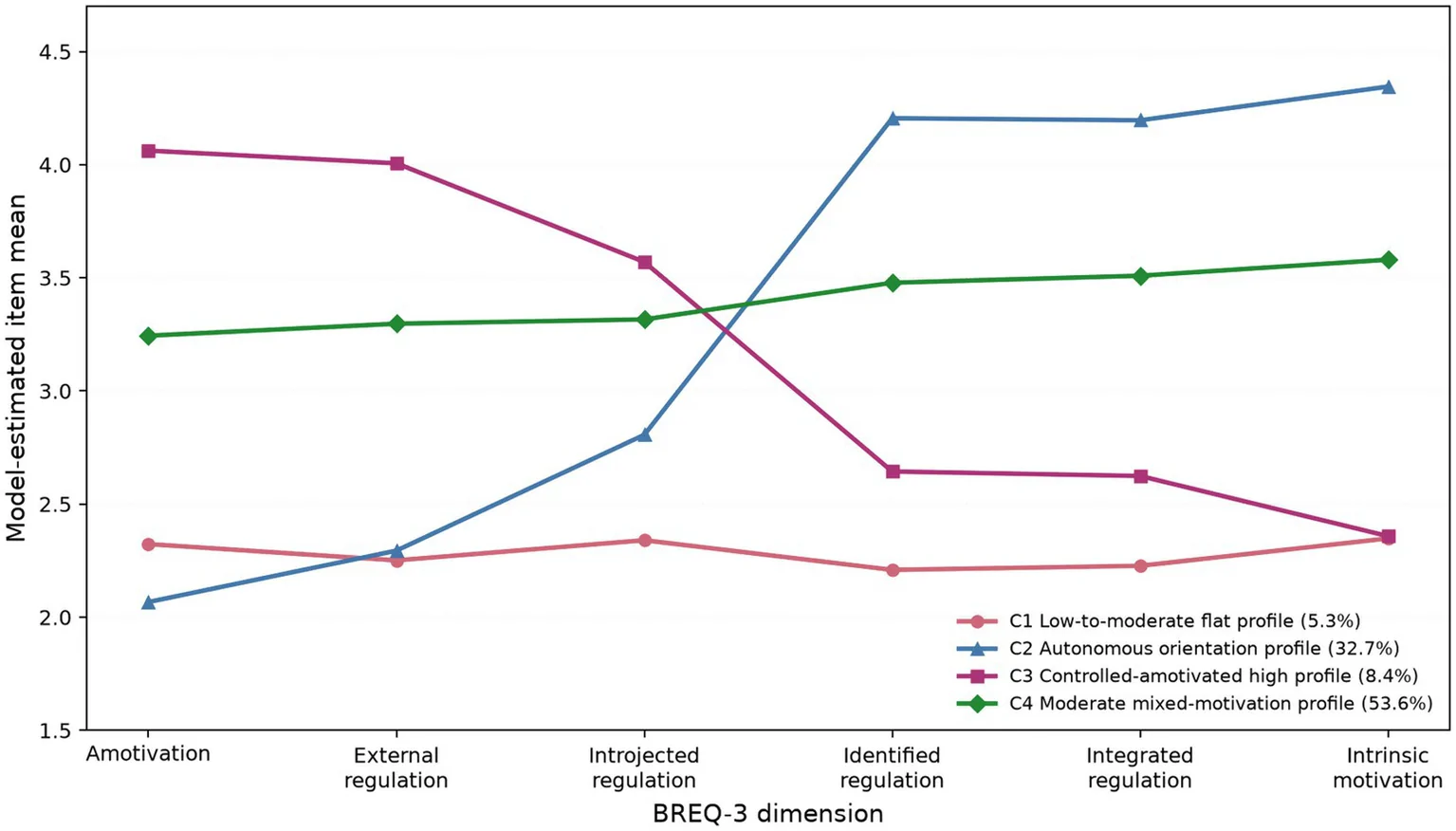
Model-estimated means of the four latent profiles of sport participation motivation among higher vocational college students.

### 3.2 Demographic differences across sport participation motivation profiles among higher vocational college students

Based on most-likely posterior probability assignment, profile membership did not differ significantly by sex, *χ*^2^ (3) = 6.599, *p* = 0.086, Cramér’s V = 0.057. A significant association was observed between grade and profile membership, *χ*^2^ (6) = 25.402, *p* < 0.001; however, the corresponding effect size was small (Cramér’s V = 0.079), indicating that the overall grade-related difference was modest. Examination of the adjusted standardized residuals showed that first-year students were overrepresented in the Autonomous Orientation Profile (4.067) and underrepresented in the Moderate Mixed-Motivation Profile (−2.672). Second-year students were underrepresented in the Autonomous Orientation Profile (−2.013) and overrepresented in the Moderate Mixed-Motivation Profile (2.076). Third-year students were underrepresented in the Autonomous Orientation Profile (−2.425) and overrepresented in the High Controlled–Amotivation Profile (3.102). No other cells showed adjusted standardized residuals exceeding |1.96|. Detailed results are presented in Tables 4, 5.

**Table 4:** Comparison of sport participation motivation profiles across demographic groups (*n* = 2,025).

Variable	Group	Total	Low-to-Moderate Flat	Autonomous Orientation	High Controlled–Amotivation	Moderate Mixed-Motivation	*χ* ^2^	*p*
Sex	Male	905	54 (6.0)	273 (30.2)	84 (9.3)	494 (54.6)	6.599	0.086
	Female	1,120	53 (4.7)	390 (34.8)	86 (7.7)	591 (52.8)		
Grade	First-year	839	40 (4.8)	317 (37.8)	62 (7.4)	420 (50.1)	25.402	<0.001
	Second-year	672	42 (6.3)	200 (29.8)	48 (7.1)	382 (56.8)		
	Third-year	514	25 (4.9)	146 (28.4)	60 (11.7)	283 (55.1)		

**Table 5:** Adjusted standardized residuals for the cross-tabulation of grade and latent profile membership.

Grade	Low-to-Moderate Flat	Autonomous Orientation	High Controlled–Amotivation	Moderate Mixed-Motivation
First-year	−0.874	4.067	−1.372	−2.672
Second-year	1.369	−2.013	−1.432	2.076
Third-year	−0.493	−2.425	3.102	0.778

Values are adjusted standardized residuals. Absolute values greater than 1.96 indicate that the observed cell frequency differs from the expected frequency at *p* < 0.05. Positive values indicate overrepresentation, whereas negative values indicate underrepresentation.

Profile membership in these descriptive cross-tabulations was determined using the most-likely posterior probability classification. Regression analyses involving auxiliary variables were conducted separately using the R3STEP procedure to account for classification uncertainty.

### 3.3 Measurement properties and Intercorrelations of campus sports culture and sport participation motivation

The four dimensions of campus sports culture were moderately correlated with one another (r = 0.342–0.608). VIF values ranged from 1.535 to 2.103 when the four dimensions were considered simultaneously, providing no indication of serious multicollinearity. The four CSC dimensions were therefore retained together in Model 1 so that their associations with latent profile membership could be evaluated while mutually adjusted. The fit indices for the four-factor confirmatory factor analysis (CFA) are reported in Section 1.2.1 (Table 6).

**Table 6:** Descriptive statistics, correlations, and VIFs for campus sports culture dimensions (*n* = 2,025).

Dimension	M	SD	1	2	3	VIF
1. Material culture	14.41	3.61	–			1.535
2. Institutional culture	14.10	3.51	0.576	–		1.952
3. Spiritual culture	14.42	3.53	0.431	0.604	–	2.103
4. Behavioral culture	14.30	3.54	0.342	0.407	0.608	1.607

Associations between campus sports culture and latent profile membership were examined using the R3STEP three-step procedure, which accounts for uncertainty in latent profile classification. The Low-to-Moderate Flat Profile was used as the reference category. In Model 1, sex, grade, and the four dimensions of campus sports culture were entered simultaneously. After adjustment for the other variables, membership in the Autonomous Orientation Profile was positively associated with material culture (OR = 1.203, 95% CI: 1.098–1.317), spiritual culture (OR = 1.311, 95% CI: 1.174–1.463), and behavioral culture (OR = 2.289, 95% CI: 2.001–2.619). Membership in the High Controlled–Amotivation Profile was positively associated only with material culture (OR = 1.204, 95% CI: 1.099–1.320). Membership in the Moderate Mixed-Motivation Profile was positively associated with material culture (OR = 1.175, 95% CI: 1.090–1.267), spiritual culture (OR = 1.109, 95% CI: 1.010–1.217), and behavioral culture (OR = 1.464, 95% CI: 1.315–1.629). Institutional culture was not significantly associated with any of the three profiles relative to the reference profile. In Model 2, sex, grade, and the total campus sports culture score were entered simultaneously. After adjustment for sex and grade, each one-point increase in the total CSC score was associated with higher odds of membership in the Autonomous Orientation Profile, High Controlled–Amotivation Profile, and Moderate Mixed-Motivation Profile rather than the Low-to-Moderate Flat Profile, with ORs of 1.287, 1.092, and 1.139, respectively (all *p* < 0.001). See Table 7.

**Table 7:** R3STEP estimates of the associations between campus sports culture and latent profile membership.

Model/Variable	Autonomous Orientation Profile *β*; OR (95% CI)	High Controlled–Amotivation Profile *β*; OR (95% CI)	Moderate Mixed-Motivation Profile *β*; OR (95% CI)
Model 1: Female (reference: male)	−0.627; 0.534 (0.275–1.038)	−0.407; 0.666 (0.325–1.364)	−0.061; 0.941 (0.523–1.692)
Model 1: Second-year (reference: first-year)	−0.427; 0.653 (0.305–1.394)	0.575; 1.778 (0.848–3.726)	0.215; 1.240 (0.634–2.425)
Model 1: Material culture	0.185; 1.203 (1.098–1.317)^***^	0.186; 1.204 (1.099–1.320)^***^	0.161; 1.175 (1.090–1.267)^***^
Model 1: Institutional culture	−0.041; 0.960 (0.868–1.061)	0.033; 1.034 (0.925–1.155)	−0.027; 0.973 (0.897–1.056)
Model 1: Spiritual culture	0.270; 1.311 (1.174–1.463)^***^	0.061; 1.063 (0.944–1.197)	0.103; 1.109 (1.010–1.217)^*^
Model 1: Behavioral culture	0.828; 2.289 (2.001–2.619)^***^	0.098; 1.103 (0.978–1.245)	0.381; 1.464 (1.315–1.629)^***^
Model 2: Total campus sports culture score	0.253; 1.287 (1.247–1.330)^***^	0.088; 1.092 (1.060–1.124)^***^	0.130; 1.139 (1.109–1.170)^***^

The Low-to-Moderate Flat Profile was the reference category. Estimates were obtained using the R3STEP procedure to account for classification uncertainty. ORs represent the relative change in the odds of profile membership associated with a one-point increase in the corresponding original, unstandardized predictor score. ^*^p < 0.05, ^***^p < 0.001.

## 4 Discussion

Using latent profile analysis (LPA), this study identified four distinct profiles of sport participation motivation among higher vocational college students: the Low-to-Moderate Flat Profile, Autonomous Orientation Profile, High Controlled–Amotivation Profile, and Moderate Mixed-Motivation Profile. The Moderate Mixed-Motivation Profile accounted for the largest proportion of the sample (52.6%), followed by the Autonomous Orientation Profile (33.1%), whereas the High Controlled–Amotivation Profile (8.4%) and Low-to-Moderate Flat Profile (5.8%) were less prevalent. These findings reveal marked heterogeneity in students’ motivational configurations. More than half of the students showed moderate levels across both controlled and autonomous forms of motivation, suggesting that multiple motivational regulations commonly coexist rather than occurring in isolation. At the same time, approximately one third of the sample exhibited a clear autonomous orientation, characterized by relatively high identified, integrated, and intrinsic motivation. These results extend conventional mean-level findings by demonstrating that sport participation motivation among higher vocational college students is better understood as a set of distinct within-person configurations.

The predominance of the Moderate Mixed-Motivation Profile is noteworthy. Students in this group showed mid-range scores across all six motivational regulations, with identified, integrated, and intrinsic motivation somewhat higher than amotivation and external regulation. This pattern is consistent with previous person-centered research showing that autonomous and controlled regulations can coexist within the same individual rather than forming mutually exclusive motivational states (Emm-Collison et al., 2020; Lindwall et al., 2017). Among students in the present sample, this profile may reflect a pattern in which personally endorsed reasons for sport coexist with external demands or social expectations. Importantly, the cross-sectional data do not indicate that this profile represents a transitional stage or that students will necessarily move toward a more autonomous pattern over time. From a practical perspective, greater choice, opportunities to experience competence, and enjoyable forms of participation may be relevant considerations for profile-informed sport provision, although their effectiveness requires further evaluation.

This study also found significant grade-level differences in sport participation motivation profiles, consistent with previous research (Ahsan et al., 2024). The distribution of motivational profiles also differed across grade levels, although the overall effect size was small (Cramér’s V = 0.079). First-year students were overrepresented in the Autonomous Orientation Profile and underrepresented in the Moderate Mixed-Motivation Profile. Second-year students showed the opposite pattern, with fewer students than expected in the Autonomous Orientation Profile and more in the Moderate Mixed-Motivation Profile. Among third-year students, the Autonomous Orientation Profile was underrepresented, whereas the High Controlled–Amotivation Profile was overrepresented. These differences are broadly consistent with previous evidence that exercise motives vary across student groups and stages of university life (Ahsan et al., 2024; Egli et al., 2011). Greater exposure to campus physical education, student clubs, and organized sport activities during the first year may coincide with more self-endorsed reasons for participation (Khajornsilp et al., 2021; Lackman et al., 2015). By contrast, internships, academic demands, and employment preparation become more prominent in later years and may be associated with a greater presence of mixed or externally regulated motives (Brown et al., 2024; Racette et al., 2008). Because these data are cross-sectional, however, the grade differences should not be interpreted as evidence that individual students become less autonomously motivated as they progress through college. Within higher vocational college settings comparable to those sampled in this study, these findings suggest that sport programs may need to take into account the different academic and practical demands faced by students at each grade level.

Campus sports culture was associated with motivational profile membership, broadly in line with previous research linking supportive sport environments with exercise motivation and participation (Tao and Yu, 2025; Zhang and Miao, 2024; Zhao et al., 2023). With the Low-to-Moderate Flat Profile as the reference category, higher overall CSC scores were associated with greater odds of belonging to each of the other three profiles. The association was strongest for the Autonomous Orientation Profile (OR = 1.287), followed by the Moderate Mixed-Motivation Profile (OR = 1.139) and the High Controlled–Amotivation Profile (OR = 1.092). This pattern indicates that students reporting a more developed campus sports culture were more likely to show stronger or more differentiated motivational engagement than a relatively flat, low-to-moderate motivational pattern. From the perspective of self-determination theory, the stronger association with the Autonomous Orientation Profile is consistent with the broader proposition that supportive social contexts may be related to more self-endorsed forms of regulation, potentially in ways that align with autonomy, competence, and relatedness (Rogowska and Morouço, 2024; Teixeira et al., 2012). The present findings, however, describe concurrent associations and do not establish that CSC produces or increases autonomous motivation. The dimension-specific results provide a more differentiated picture. Material culture was positively associated with all three profiles relative to the Low-to-Moderate Flat Profile, with ORs ranging from 1.175 to 1.204. This relatively broad pattern suggests that access to facilities, equipment, and activity spaces may distinguish students with more evident motivational engagement from those showing a flatter motivational pattern, although material resources alone do not appear to distinguish the quality of motivation particularly strongly (Chaabna et al., 2022; Ziegler, 2024). Behavioral culture showed the largest association observed in Model 1: each one-point increase was associated with greater odds of membership in the Autonomous Orientation Profile (OR = 2.289) and, to a lesser extent, the Moderate Mixed-Motivation Profile (OR = 1.464), whereas its association with the High Controlled–Amotivation Profile was not statistically significant. Spiritual culture followed a similar but weaker pattern, being associated with the Autonomous Orientation Profile (OR = 1.311) and Moderate Mixed-Motivation Profile (OR = 1.109), but not with the High Controlled–Amotivation Profile. These findings are consistent with the possibility that an active participation climate and stronger recognition of sport value are more closely aligned with motivational configurations containing greater autonomous regulation (Leary et al., 2023; Motevalli et al., 2024; Su and Liu, 2025; Zhou et al., 2025). By contrast, institutional culture was not independently associated with any profile after the other CSC dimensions were considered simultaneously. This null finding should not be interpreted as evidence that institutional arrangements are unimportant; rather, formal rules and management structures did not show a unique association with profile membership beyond the material, spiritual, and behavioral dimensions measured in this study. The profile-specific findings may also generate hypotheses for future research on differentiated sport provision, although they should not be interpreted as evidence of intervention effectiveness. Because the present study did not assess subsequent physical activity behavior or physical and mental health outcomes, any practical implications remain tentative. For students showing the Low-to-Moderate Flat Profile, future research could examine whether accessible, enjoyable, and low-threshold activities are associated with greater willingness to participate. For those in the High Controlled–Amotivation Profile, longitudinal or intervention studies could test whether environments characterized by greater choice, competence-supportive feedback, and personally meaningful reasons for participation are related to more self-endorsed forms of regulation. Among students in the Moderate Mixed-Motivation Profile, future studies could examine whether varied activity options, peer support, and constructive feedback are associated with the maintenance or quality of sport participation. For students in the Autonomous Orientation Profile, clubs, competitions, volunteer activities, leadership opportunities, and skill-development programs may represent relevant contexts for examining the persistence of autonomous motivation and sport engagement. These possibilities should be evaluated in prospective or intervention studies that include objective indicators of physical activity and relevant physical and mental health outcomes.

## 5 Limitations

Several limitations should be noted. First, the cross-sectional design limits causal inference regarding the relationship between campus sports culture (CSC) and sport participation motivation profiles. Future studies should use longitudinal or intervention designs to examine their reciprocal changes over time. Second, the sample was drawn through convenience sampling from three higher vocational colleges in Jiangsu and Zhejiang Provinces. The findings should therefore not be regarded as representative of higher vocational college students in China as a whole and are most appropriately interpreted in relation to the sampled colleges and institutions with broadly comparable student populations and educational contexts. Future studies using probability-based sampling and a wider range of regions and institution types are needed to assess the generalizability of the identified motivational profiles and their associations with campus sports culture. Third, reliance on self-reported questionnaires may introduce common method bias and social desirability effects. Future research could combine objective physical activity indicators, interview data, and institutional records of campus sport participation. Finally, future studies should consider factors such as exercise self-efficacy, peer support, perceived occupational physical demands, and school belonging to clarify the pathways through which CSC may be linked to different sport participation motivation profiles.

## 6 Conclusion

Using latent profile analysis, this study identified four distinct configurations of sport participation motivation among higher vocational college students: the Low-to-Moderate Flat Profile, Autonomous Orientation Profile, High Controlled–Amotivation Profile, and Moderate Mixed-Motivation Profile. The Moderate Mixed-Motivation Profile was the most prevalent, followed by the Autonomous Orientation Profile, indicating that multiple forms of motivational regulation commonly coexist within this population. Campus sports culture was associated with profile membership, with the overall association being strongest for the Autonomous Orientation Profile. At the dimensional level, material culture was associated with all three non-reference profiles, whereas behavioral and spiritual culture showed more selective associations with the Autonomous Orientation and Moderate Mixed-Motivation Profiles; institutional culture showed no independent association. Within the three sampled colleges, these findings suggest that motivational heterogeneity and different aspects of the campus sport environment may be relevant considerations when developing profile-informed physical activity programs, although the effectiveness of such approaches remains to be tested. Similar implications may be relevant to higher vocational colleges with comparable student populations and institutional contexts in Jiangsu and Zhejiang Provinces, although broader generalization requires further evidence. Because the study was cross-sectional, the observed relationships should be interpreted as associations rather than evidence of causal effects.

## Data Availability

The raw data supporting the conclusions of this article will be made available by the authors, without undue reservation.
